# Protein C Gene Mutation in an Older Adult Patient with *Clostridium perfringens* Septicemia-Related Visceral Vein Thrombosis

**DOI:** 10.1055/s-0041-1728664

**Published:** 2021-05-26

**Authors:** Kiyoko Kanosue, Satomi Nagaya, Eriko Morishita, Masayoshi Yamanishi, Shinsaku Imashuku

**Affiliations:** 1 Department of Internal Medicine, Uji-Tokushukai Medical Center, Uji, Japan; 2 Department of Clinical Laboratory Science, Kanazawa University Graduate School of Medical Science, Kanazawa, Japan; 3 Department of Laboratory Medicine, Uji-Tokushukai Medical Center, Uji, Japan

**Keywords:** visceral vein thrombosis, protein C deficiency, *Clostridium perfringens*, septicemia, older adult

## Abstract

A 78-year-old Japanese male with *Clostridium perfringens* septicemia and cholecystitis was found to have thrombosis in the left branch of intrahepatic portal vein as well as superior mesenteric vein. Visceral vein thrombosis (VVT) in this case was associated with protein C deficiency, due to a heterozygous mutation, p. Arg185Met. Our experience emphasizes that VVT, or other thromboembolic events, may occur in later life, triggered by environmental thrombosis risk factors, together with underlying hereditary protein C gene mutation.

## Introduction

Thrombophilia, whether acquired or hereditary, is linked to the development of visceral vein thrombosis (VVT). 1 Hereditary thrombophilia, including deficiencies of antithrombin, protein C, and protein S, is a major cause of venous thromboembolism in pregnancy as well as idiopathic thromboembolism, in young or middle-aged patients. 2 Heterozygous lesions of the *PROC* gene have been noted in symptomatic younger adults with protein C deficiency (30–65% activity), 3 while reports of hereditary protein C deficiency are limited in older adult patients. Here, we report a heterozygous *PROC* gene mutation in a case of *Clostridium perfringens* septicemia-related VVT in an older adult.

## Case Report

**Fig. 1:**
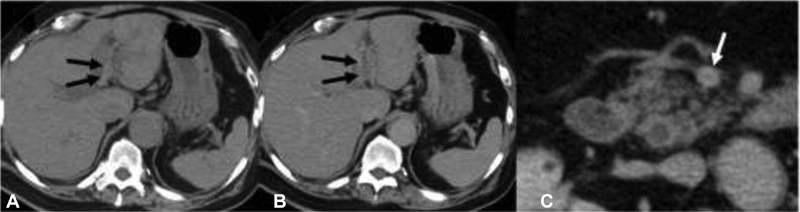
Computed tomography (CT) showing thrombus ( *arrows* ) in the left branch of the intrahepatic portal vein ( **A** , noncontrast and **B** , contrast images) as well as in the superior mesenteric vein ( **C** , contrast image).

A 78-year-old male was referred to our clinic with fever and abdominal pain. Abdominal computed tomography (CT) and ultrasound scan 1 month prior to his referral and admission revealed no abnormalities. The patient had been administered oral linagliptin (DDP-4 inhibitor), amlodipine, clopidogrel, atorvastatin, and rabeprazole for the management of his diabetes mellitus/hypertension/hyperlipidemia, as well as for prevention of ischemic cerebrovascular disease. He was a former smoker (two packs per day) but had quit 1 year earlier. He was not a heavy drinker. On admission, he was alert, with blood pressure 94/64 mm Hg, heart rate 141/min, respiratory rate 28/min, and SpO _2_ 93% (under oxygen, 3 L/min). He was suspected to have cholecystitis, based on the abdominal CT image after admission and following laboratory data: white blood cells, 14,900/µL; hemoglobin, 14.6 g/dL; platelet count, 154 K/µL. Inflammatory markers were significantly elevated with serum C-reactive protein, 11.19 (reference; <0.29) mg/dL and procalcitonin, 21.17 (<0.4) mg/dL. He also showed diabetic data with blood glucose, 255 (70–110) mg/dL and HbA1C, 12.7 (3.8–6.2)%. Hepatic function was abnormal with aspartate aminotransferase, 352 (13–37) U/L; alanine aminotransferase, 335 (8–45) U/L; lactate dehydrogenase, 580 (122–228) U/L; gamma-glutamyl transpeptidase, 463 (12–49) U/L; total bilirubin, 4.79 (0.3–1.3) mg/dL; hyaluronic acid, 287 (<50) ng/mL, and type 4 collagen, 339 (<140) ng/mL, but with normal total protein and albumin/globin ratio. Renal and cardiac functions were slightly abnormal with serum blood urea nitrogen, 20.9 mg/dL and creatinine, 2.06 (0.64–1.11) mg/dL, and troponin I was slightly elevated with 0.151 (<0.03) ng/mL. Blood culture yielded *C. perfringens* . Abdominal ultrasound scan revealed an enlarged gall bladder, as well as loss of blood circulation signal in the left branch of the intrahepatic portal vein. These findings as well as superior mesenteric vein thrombosis was confirmed by abdominal CT ( Fig. 1 ), indicating the presence of VVT. As summarized in Table 1 , coagulation/fibrinolysis status analysis demonstrated that the patient had protein C deficiency, both activity as well as antigen. DIC (disseminated intravascular coagulation) was ruled out. Although troponin I levels were slightly high, myocardial infarction was excluded. Also, gastrointestinal tract malignancies were ruled out by endoscopy and normal levels of tumor markers. The patient was diagnosed with cholangitis-related *Clostridium* sepsis associated with VVT and treated successfully with meropenem/vancomycin antibiotics and continuous intravenous heparin. At 2 weeks from admission, heparin was switched to oral warfarin and the patient was discharged 3 weeks later with persistent VVT. During his hospital stay, protein C activity was assayed five times, with results ranging from of 30 to 59% (reference; 70–140%). We also confirmed the persistent protein C deficiency (49%), 6 months after acute episode of thrombosis, when he showed normal protein S (67.2%) and antithrombin (84%) activity. These data support that he has had inherent protein C deficiency, not due to consumption associated with thrombotic event. Thus, we conducted mutation analysis of the *PROC* gene and identified the following heterozygous mutation in exon 7: c.554G > T, AGG > ATG, p. Arg185Met.

**Table 1:** Summary of coagulation/fibrinolysis and autoimmune studies

Factor (reference)	Measurement
PT-INR (0.9–1.1)	1.24
APTT (relative index)	0.968
DIC score (>6) a	3
D-dimer (<1.0) µg/mL	42.0 µg/mL
TAT (<4.0) ng/mL	16.4 ng/mL
PIC (<0.8) µg/mL	3.1 µg/mL
MPO-ANCA (<0.5) IU/mL	<0.5 IU/mL
PR3-ANCA (<0.5) IU/mL	<0.5 IU/mL
Protein C activity (70–100%) b	42%
Protein C antigen (70–150%) b	30%
Protein S activity (63.5–149%)	82.1%
Antithrombin activity (80–130%)	75%
ACL-β2GP1 (<3.4) U/mL	<1.3 U/mL
ACL-IgG (<9) U/mL	3 U/mL
LAC (SCT; <1.16) s	0.67 s
Homocysteine (6.3–18.9) mmol/mL	11.4 mmol/mL
ANA (<40)	<40

Abbreviations: ACL, anticardiolipin; ANA, antinuclear antibody; ANCA, antineutrophil cytoplasmic antibody; APTT, activated partial thromboplastin time; IgG, immunoglobulin G; INR, international normalized ratio; LAC, lupus anticoagulant; MPO, myeloperoxidase; NT, not tested; PIC, plasmin α2 plasmin inhibitor complex; PR3, proteinase 3; PT, prothrombin time; SCT, silica clotting time; TAT, thrombin-antithrombin complex; β2GP1, β _2_ -glycoprotein 1.

a Based on Wada H, Takahashi H, Uchiyama T, Eguchi Y. The approval of revised diagnostic criteria for DIC from the Japanese Society on Thrombosis and Hemostasis. Thrombosis J 2017;15:17.

b Protein C activity was assayed by synthetic substrate method and protein C antigen by latex agglutination method.

## Discussion

Several conditions known to cause VVT, 4 5 including hepatic cirrhosis, pancreatitis, and malignancies, were ruled out in our case; however, the patient had *Clostridium* sepsis, and diabetes mellitus. 6 We first thought that his VVT was due to a pathological condition similar to thrombophlebitis of the portal vein, caused by *C. perfringens* septicemia 7 ; however, we discovered that probably inherent protein C deficiency was also involved in the development of VVT.

Hereditary protein C deficiency is caused by mutation of the *PROC* gene, located on chromosome 2q14.3, which consists of nine exons, with heterozygous mutations more common than homozygous changes. 1 8 Defects in various exons of the *PROC* gene have been reported among Japanese families with protein C deficiency. 2 9 10 The p. Arg185Met mutation (numbering based on current notation) identified in this case appears to be a novel amino acid substitution and pathogenic. Our sequence homology search for Arg185 and adjacent amino acids revealed a highly conserved region among seven mammals ( Supplementary Fig. S1 ) indicating that Arg185 is important for the expression of normal function of protein C. Furthermore, the steric structure analysis of protein C ( Supplementary Fig. S2 ) suggests that p. Arg185Met may disrupt the conformation of protein C and affect its intracellular degradation or stability after secretion. At this same site, arginine to serine substitution (p. Arg143Ser; based on previous notation) in a case of thrombosis was also previously described by Miyata et al. 11

To date, young or middle-aged adults with heterozygous changes in the *PROC* gene have been reported to develop thromboembolic symptoms, 2 while similar reports of older adult patients with hereditary protein C deficiency are rare. 12 Our experience indicates that VVT, or other thromboembolic events, may occur in later life, triggered by environmental thrombosis risk factors, together with underlying hereditary *PROC* gene mutation. Though prothrombotic gene testing is important to clarify the precise cause of thrombotic event, results may not be helpful for the management of patients. In addition, it is controversial if gene testing helps for members of their family. Therefore, *PROC* gene analyses may be considered in special and specific group of patients with persistent protein C deficiency.
