# Supplementary material for: Protein C Gene Mutation in an Older Adult Patient with Clostridium perfringens Septicemia-Related Visceral Vein Thrombosis
Source: TH Open. 2021 May 26;5(2):e171–3. doi: 10.1055/s-0041-1728664 (PMC8154515; doi:10.1055/s-0041-1728664)
Supplement: Supplementary file 1 — Supplementary Material [file 10-1055-s-0041-1728664-s210005.pdf]

Amino Acid No.									185							
Homo sapiens	•••	A	V	K	F	P	C	G	R	–	P	W	K	R	M	•••
Bos taurus	•••	K	V	T	F	P	C	G	R	L	G	–	K	R	M	•••
Oryctolagus cuniculus	•••	A	V	R	F	P	C	G	R	L	G	W	K	R	I	•••
Gallus gallus	•••	V	V	E	F	P	C	G	R	V	K	M	D	Y	–	•••
Rattus norvegicus	•••	T	V	N	F	P	C	G	K	L	–	W	K	R	T	•••
Mus musculus	•••	T	V	N	F	P	C	G	K	L	–	G	R	W	I	•••
Xenopus laevis	•••	V	V	E	F	P	C	G	K	S	K	I	V	–	–	•••

Supplementary Fig. S1 Comparison of amino acid sequence of protein C in seven species show Arg 185 in incompletely but highly conserved while the four adjacent amino acids are completely conserved. Results indicate that this region is an important site for normal protein C function. (Red letters indicate Arg185 and the conserved identical amino acids among different species in the region are in light blue boxes).

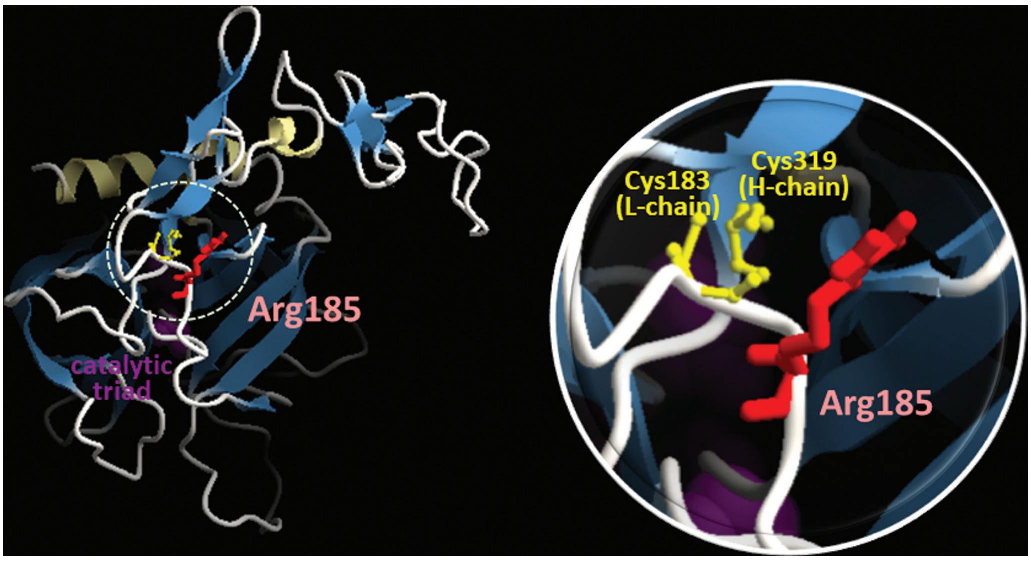

Supplementary Fig. S2 Molecular modeling of human activated protein C (PDB ID: 1AUT). Red sticks denote Arg185. The catalytic triad is composed of His-253, Asp299, and Ser402 (*purple balls*) and yellow sticks denote the disulfide bond (Cys183-Cys319). Because Arg185 is far from the catalytic triad, it is unlikely that p.Arg185Met directly affects the serine protease activity of protein C. However, since Arg185 is located close to the disulfide bond that connects the light chain and heavy chain, loss of positive charge on arginine residue due to amino acid substation may disrupt the conformation of protein C and affects its intracellular degradation or stability after secretion.

 © 2021. The Author(s).
